# Small Non-coding RNA RyhB Mediates Persistence to Multiple Antibiotics and Stresses in Uropathogenic *Escherichia coli* by Reducing Cellular Metabolism

**DOI:** 10.3389/fmicb.2018.00136

**Published:** 2018-02-06

**Authors:** Shanshan Zhang, Shuang Liu, Nan Wu, Youhua Yuan, Wenhong Zhang, Ying Zhang

**Affiliations:** ^1^Key Lab of Molecular Virology, Department of Infectious Diseases, Huashan Hospital, Fudan University, Shanghai, China; ^2^Department of Molecular Microbiology and Immunology, Bloomberg School of Public Health, Johns Hopkins University, Baltimore, MD, United States

**Keywords:** *Escherichia coli*, persisters, small RNA, RyhB, metabolism, ATP, NAD^+^/NADH, antibiotics

## Abstract

As dormant phenotypic variants of bacteria, persisters account for many chronic infections affecting human health. Despite numerous studies, the role of small non-coding RNA (sRNA) in bacterial persistence has not been reported. To investigate the role of Hfq-interacting sRNA in persistence, we constructed the deletion mutants of 20 Hfq-interacting sRNAs (RyhB, GcvB, MgrR, RybB, MicF, SgrS, RprA, DicF, SsrS, FnrS, GadY, DsrA, OmrB, ArcZ, RyeB, RydC, OmrA, MicA, MicC, and ChiX) to assess their persistence capacity in uropathogenic *Escherichia coli* strain UTI89 and identified a new sRNA RyhB being involved in persister formation. The *ryhB*-knockout mutant had significant defect in persistence to a diverse range of antibiotics (levofloxacin, cefotaxime, gentamicin) and stresses (hyperosmosis, acid, and heat) in both exponential phase and stationary phase. In addition, the effect of RyhB on persistence was synergistic with ppGpp and Fur protein. RNA-Seq analysis indicated that the *ryhB*-knockout mutant had a hyperactive metabolic state compared with the parent strain. Interestingly, increased adenosine triphosphate (ATP) levels and altered NAD^+^/NADH ratios were observed in the *ryhB*-knockout mutant. Our findings represent a new level of persistence regulation via sRNA and may provide novel therapeutic targets for interventions.

## Introduction

Persisters represent a small number of metabolically quiescent bacteria that survive exposure to bactericidal drugs and stresses while remaining susceptible to drugs and stresses under appropriate conditions (Lewis, 2010; Zhang, 2014). They are genetically identical to the other cells, but exhibit phenotypic differences (Allison et al., 2011a; Zhang, 2014). The recalcitrance of many chronic and persistent bacterial infections, such as tuberculosis, Lyme disease, urinary tract infections (UTIs), and biofilm infections is associated with the presence of persisters (Blango and Mulvey, 2010; Zhang et al., 2012; Levin et al., 2014; Zhang, 2014; Liu et al., 2016). Thus far, many studies on persistence mechanisms have been performed in *Escherichia coli* (*E. coli*), with multiple genes and pathways being identified as involved in persister formation, including toxin-antitoxins (TA), SOS response, and DNA repair, signal transduction, membrane stress, energy production, phosphate metabolism, and protein degradation (Zhang, 2014; Harms et al., 2016). However, these findings indicate that persistence is a very complex phenomenon with redundant mechanisms and that there may be other more important potential mechanisms that remain unknown.

In bacteria, small non-coding RNAs (sRNAs), which are relatively short transcripts (~50–300 nucleotides), regulate a wide range of physiological functions in response to external signals (Wassarman, 2002). sRNAs can act by modulating transcription, translation, mRNA stability, DNA maintenance, or silencing by binding to the untranslated region of target mRNAs (Chabelskaya et al., 2010; Gottesman and Storz, 2011; Sayed et al., 2011). Acting as an sRNA chaperone, the global regulator Hfq, facilitates base-pairing interactions between sRNAs. Hfq can not only function directly on its targets but also function indirectly through base-pairing of sRNAs with target mRNAs (Chao and Vogel, 2010). *hfq* mutant has been reported to have reduced persister formation capacity upon challenge with ampicillin in *E. coli* and *Salmonella enterica* (Kim and Wood, 2010; Papenfort and Vogel, 2010; Hebrard et al., 2012). However, whether the role of Hfq in persistence has anything to do with the Hfq-interacting sRNAs remains unknown.

In order to study the role of the Hfq-interacting sRNAs in persistence, we generated deletion mutants of 20 Hfq-binding sRNAs in uropathogenic *E. coli* UTI89 strain and assessed their persister phenotypes. Our results provide the first evidence of a role for sRNA in regulating persistence, with implications for developing more effective treatments for persistent infections.

## Materials and methods

### Bacterial strains and growth conditions

The bacterial strains used in this study were derivatives of the parent strain UTI89. They were routinely cultured in Luria-Bertani (LB) broth (10 g bacto-tryptone, 5 g yeast extract, and 10 g NaCl/L) at 37°C, 200 rpm. Bacterial stock stored at −80°C was transferred into fresh LB medium and grown overnight before being used for persister experiments.

### Construction of deletion mutants and overexpression strains

The deletion mutants of Hfq-dependent sRNAs were constructed using the λ Red recombination system as described by Datsenko and Wanner (2000). Primers used to amplify all knockout-DNA fragments and verify the correct constructs by polymerase chain reaction (PCR) are shown in Supplementary Tables 1, 2. To create double-deletion mutants, the chloramphenicol-resistance gene was removed from plasmid pCP20.

The arabinose-inducible plasmid pBAD202 was used to construct overexpression strains according to previous report (Ma et al., 2010). Primers (F: 5′-CATG CCATGGAAAAGCCAGCACCCGGC-3′ and R: 5′-CCCGGAATTCGCGATCAGGAAGACCCTCG-3′) used for the construction of the plasmid containing the RyhB gene were designed in this study. Genes were amplified with PCR primers, followed by digestion of both the PCR fragments and pBAD202 with the restriction enzymes *Nco*I and *Eco*RI (New England Biolabs, Ipswich, MA, USA) and ligation using the T4 DNA ligase (New England Biolabs). The new constructs along with the empty vector, pBAD202, were transformed into parent strain UTI89 for overexpression experiments. The deletion mutants and overexpression strains were verified by DNA sequencing. Arabinose (0.1%) was added to the cultures of overexpression strains to induce the conditional expression after bacterial culture for appropriate time.

### Persister assay

Persister levels were determined by counting the number of colony forming units (CFUs) that grew on LB agar plates as previously described (Ma et al., 2010; Wang et al., 2015). The antibiotics levofloxacin (5 μg/mL), cefotaxime (128 μg/mL), gentamicin (30 μg/mL) were added directly to cultures at the exponential (about 3 h of cultivation, ~10^8^ CFU/mL) or stationary (10 h of cultivation, ~10^9^ CFU/mL) phase unless otherwise stated. Aliquots of the bacterial cultures exposed to antibiotics were incubated at 37°C at different time points and washed in phosphate-buffered saline before plating on LB plates in the absence of antibiotics to determine CFU count.

### Susceptibility to various stresses

For heat shock, bacteria were placed in a water bath at 55°C for 3 h. For acid stress (pH 3.0) and hyperosmosis (NaCl, 4 M), cultures were washed twice with acid or hyperosmotic LB medium and resuspended in the same column of corresponding LB medium, respectively. Aliquots of the bacterial cultures exposed to various stresses were incubated at 37°C at different time points and washed in phosphate-buffered saline before plating on LB plates in the absence of antibiotics to determine CFU count.

### RNA isolation and real-time PCR

Bacteria used for real-time PCR (RT-PCR) analysis were routinely cultured for 10 h in LB medium, followed by centrifugation at 5,000 rpm and 4°C to remove the supernatant. RNA Protect bacteria reagent (Qiagen, Hilden, Germany) was added to resuspended cells, and total RNA was isolated from cells using a bacterial RNA kit (Omega Bio-tek, Norcross, GA, USA) according to manufacturer protocol for the real-time PCR experiment. Total RNA was converted to cDNA using the PrimeScript TMRT reagent kit with gDNA Eraser (Takara, Shiga, Japan) according to manufacturer protocol and used as a template to perform real-time PCR on an Applied Biosystems 7500 real-time instrument (Applied Biosystems, Foster City, CA, USA). The primers for real-time PCR are listed in Supplementary Table 3. The 16S rRNA gene *rrsB* was used as the reference gene, and changes in expression were presented as the average of three biological replicates.

### RNA-sequencing

RNA extraction, quality assessment, sequencing, and analysis were performed by Shanghai Biotechnology Corporation (Shanghai, China) using method described previously (Xu et al., 2016). Briefly, total RNA was extracted using an RNeasy mini kit (Qiagen) according to manufacturer protocol, and the RNA integrity number was assessed using an Agilent Bioanalyzer 2100 (Agilent Technologies, Santa Clara, CA, USA). Qualified total RNA was further purified using an RNA clean XP kit (Beckman Coulter, Brea, CA, USA) and a RNase-free DNase set (Qiagen). rRNA removal, fragmentation, synthesis of the second strand, adenylation of 3′ ends, adapter ligation, and amplification were prepared prior to sequencing using an Illumina HiSeq 2500 (Illumina, San Diego, CA, USA). Reads were assessed and quantified before mapping to genome of *E. coli* UTI89.

### Determination of intracellular ATP concentration

Intracellular ATP concentration was determined according to method described previously (Shan et al., 2017). At the appropriate time points, the BacTiter-Glo microbial cell viability assay kit (Promega) was used following the recommended protocol. Briefly, 100 μL BacTiter-Glo reagent and 100 μL cell-culture medium were mixed in 96-well plates on an orbital shaker and incubated for 5 min, followed by determination of luminescence using the SpectraMax Paradigm multi-mode detection platform (Applied Biosystems). The number of bacteria in the 100 μL cell-culture was also determined at the corresponding time. After that, the luminescence of a single cell was calculated.

### Measurement of intracellular NAD^+^ and NADH concentration

Intracellular NAD^+^ and NADH concentrations were measured according to methods described previously (Vilcheze et al., 2005; Maeda et al., 2017). For NAD^+^ and NADH extraction, bacteria were cultured to the designed timepoints and harvested by centrifugation at 14,000 rpm at 4°C for 3 min. Using the NAD^+^/NADH Assay Kit (BioAssay Systems, Hayward, CA, USA), the extraction procedure and measurement were performed according to the manufacturer's instructions. The reaction was performed in flat-bottom 96-well plates. The NAD^+^/NADH concentration ratio was calculated based on the measured values of OD_565_.

## Results

### Identification of a new persister sRNA, RyhB

To explore the role of sRNA in persistence, we constructed the knockout-mutant strains of 20 known Hfq-binding sRNAs (RyhB, GcvB, MgrR, RybB, MicF, SgrS, RprA, DicF, SsrS, FnrS, GadY, DsrA, OmrB, ArcZ, RyeB, RydC, OmrA, MicA, MicC, and ChiX; Mandin and Gottesman, 2010; Kim et al., 2015) in *E. coli* UTI strain UTI89 and exposed the mutants to bactericidal antibiotic levofloxacin (5 μg/mL) in the stationary phase (cultured for 10 h, ~10^9^ CFU/mL). Among these mutants, the RyhB knockout mutant strain Δ*ryhB* displayed a dramatic decrease in persister levels compared with the parent strain UTI89. Thus, RyhB was selected for further investigation below.

### Susceptibilities of the Δ*ryhB* mutant to a variety of antibiotics

**Figure 1:**
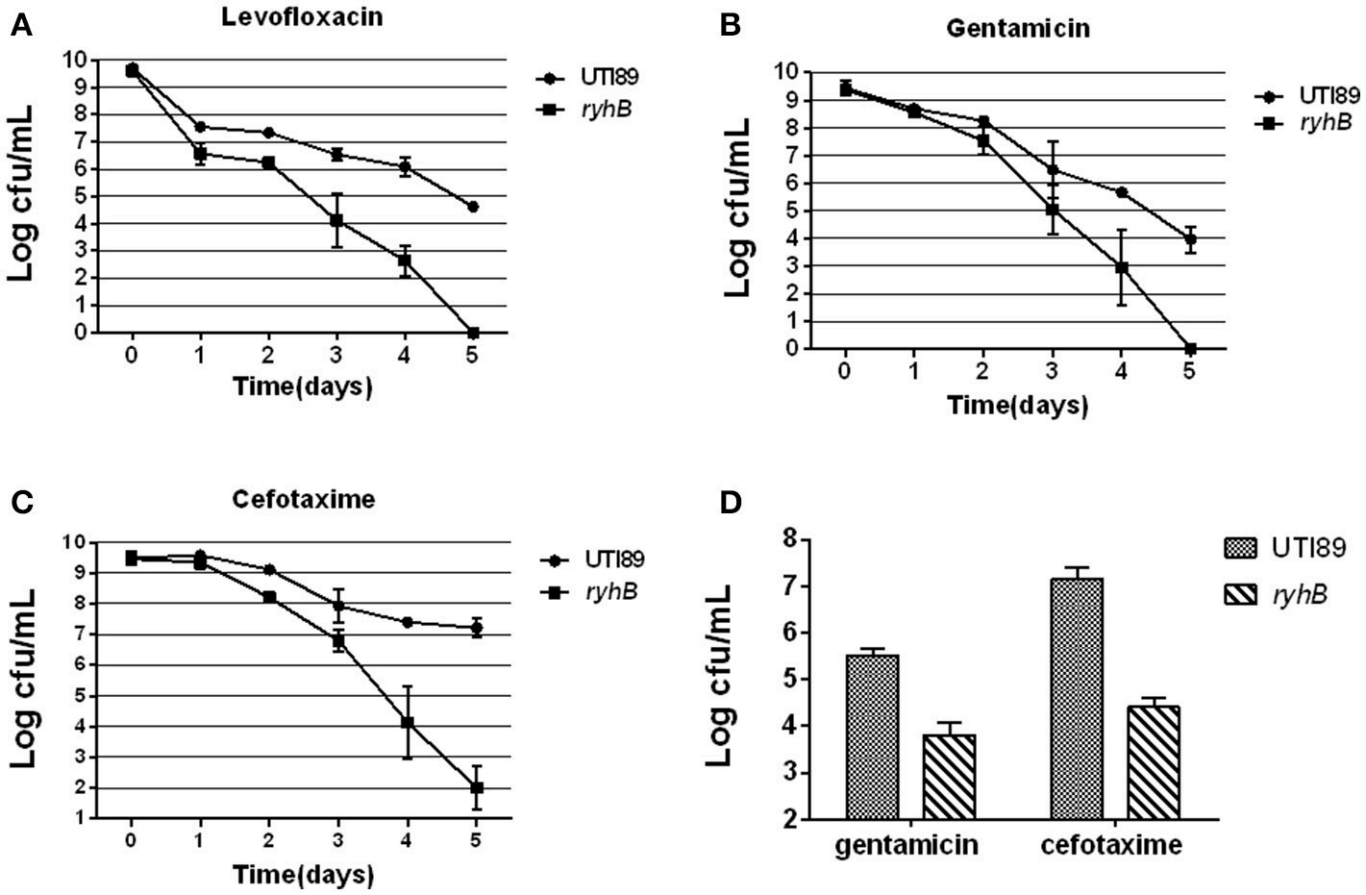
Susceptibilities of the Δ*ryhB* mutant to a variety of antibiotics. **(A–C)**. Susceptibilities of the stationary phase bacteria (~10^9^ CFU/mL) were determined every day after exposure to levofloxacin (5 μg/mL, **A**), gentamicin (30 μg/mL, **B**) or cefotaxime (128 μg/mL, **C**). **(D)** Susceptibilities of the exponential phase bacteria (~10^8^ CFU/mL) were determined after exposure to gentamicin (30 μg/mL) or cefotaxime (128 μg/mL) for 6 h. Error bars show standard variations in CFU between at least three independent experiments.

To better understand the role of RyhB in persistence, dynamic responses of the *ryhB* knockout mutant strain Δ*ryhB* and the parent strain UTI89 upon exposure to antibiotics (levofloxacin, cefotaxime, gentamicin) were examined. In the stationary phase (cultured for 10 h, ~10^9^ CFU/mL), the Δ*ryhB* mutant showed ~10^5^-fold lower persister level than the parent strain UTI89 upon treatment with levofloxacin (5 μg/mL) for 5 days (Figure 1A). The case was also applicable to gentamicin and cefotaxime. After gentamicin (30 μg/mL) or cefotaxime (128 μg/mL) treatment for 5 days, the Δ*ryhB* mutant also had lower persister numbers than UTI89, which had a ~10^4^- and ~10^5^-fold decrease, respectively (Figures 1B,C).

Because persisters are heterogeneous, and the age of the bacterial culture can affect persister level (Li and Zhang, 2007; Luidalepp et al., 2011; Zhang, 2014), we determined whether RyhB influenced persister level in the exponential phase. Bacteria were cultured for about 3 h to reach the density of ~10^8^ CFU/mL before antibiotics were added. Following gentamicin (30 μg/mL) or cefotaxime (128 μg/mL) exposure, we found the survival of the Δ*ryhB* mutant was decreased (~10^2^- and ~10^3^-fold, respectively) compared with the parent strain (Figure 1D). These results indicate that RyhB was indeed involved in persistence to a wide range of antibiotics in the exponential phase, as well as in the stationery phase.

Furthermore, the Δ*ryhB* mutant displayed a similar defect in persister-formation capacity during the early stationary phase (cultured for 6 h, ~10^9^ CFU/mL) as well as late stationary phase (cultured for 24 h, ~10^9^ CFU/mL) (data not shown).

### Hypersensitivities of the Δ*ryhB* mutant to stresses

**Figure 2:**
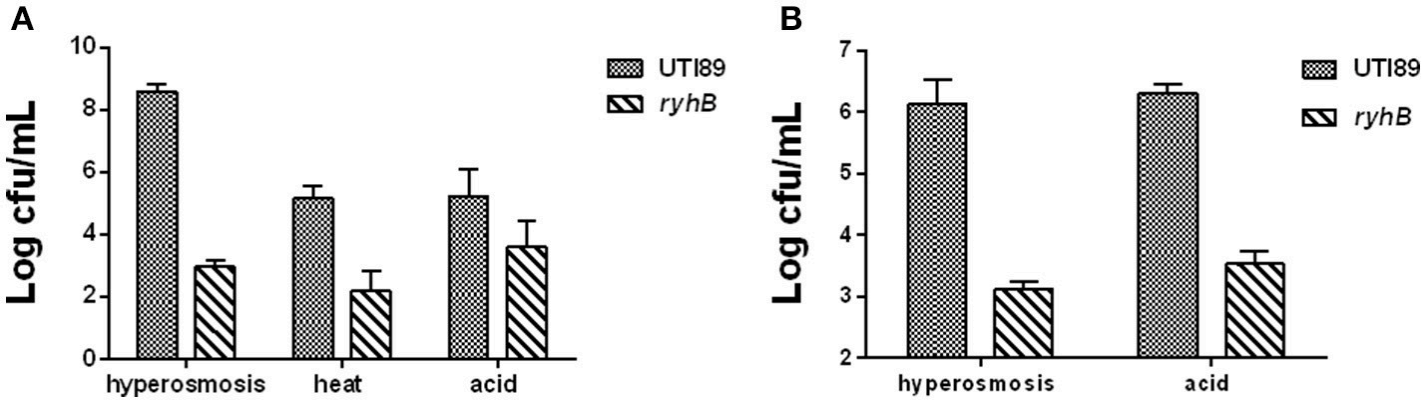
Hypersensitivities of the Δ*ryhB* mutant to stresses. **(A)** Susceptibilities of the stationary phase bacteria (~10^9^ CFU/mL) were determined after exposure to hyperosmosis (NaCl, 4 M) for 3 d, heat (55°C) for 3 h or acid (pH 3.0) for 12 h. **(B)** Susceptibilities of the exponential phase bacteria (~10^8^ CFU/mL) were determined after exposure to hyperosmosis (NaCl, 4 M) or acid (pH 3.0) for 3 h. Error bars show standard variations in CFU between at least three independent experiments.

To test the effect of stress on the Δ*ryhB* mutant, we subjected the Δ*ryhB* mutant and the parent strain UTI89 to various stresses (hyperosmosis, acid, and heat). In the stationary phase (cultured for 10 h, ~10^9^ CFU/mL), we observed a ~10^5^-fold lower survival of the Δ*ryhB* mutant than the parent strain UTI89 under hyperosmosis (NaCl, 4 M) exposure. In addition, the Δ*ryhB* mutant was also more sensitive to other stresses including heat (55°C) and acid (pH 3.0), with a ~10^3^-fold lower survival to heat, and a ~10^2^-fold lower survival to acid, respectively (Figure 2A).

In addition, the effect of stress on the Δ*ryhB* mutant could also be observed in the exponential phase (cultured for about 3 h, ~10^8^ CFU/mL). During hyperosmosis (NaCl, 4 M) or acid (pH 3.0) exposure, the Δ*ryhB* mutant had ~10^3^-fold lower persister numbers than the parent strain UTI89 (Figure 2B).

### Overexpression of RyhB confers higher persistence levels

**Figure 3:**
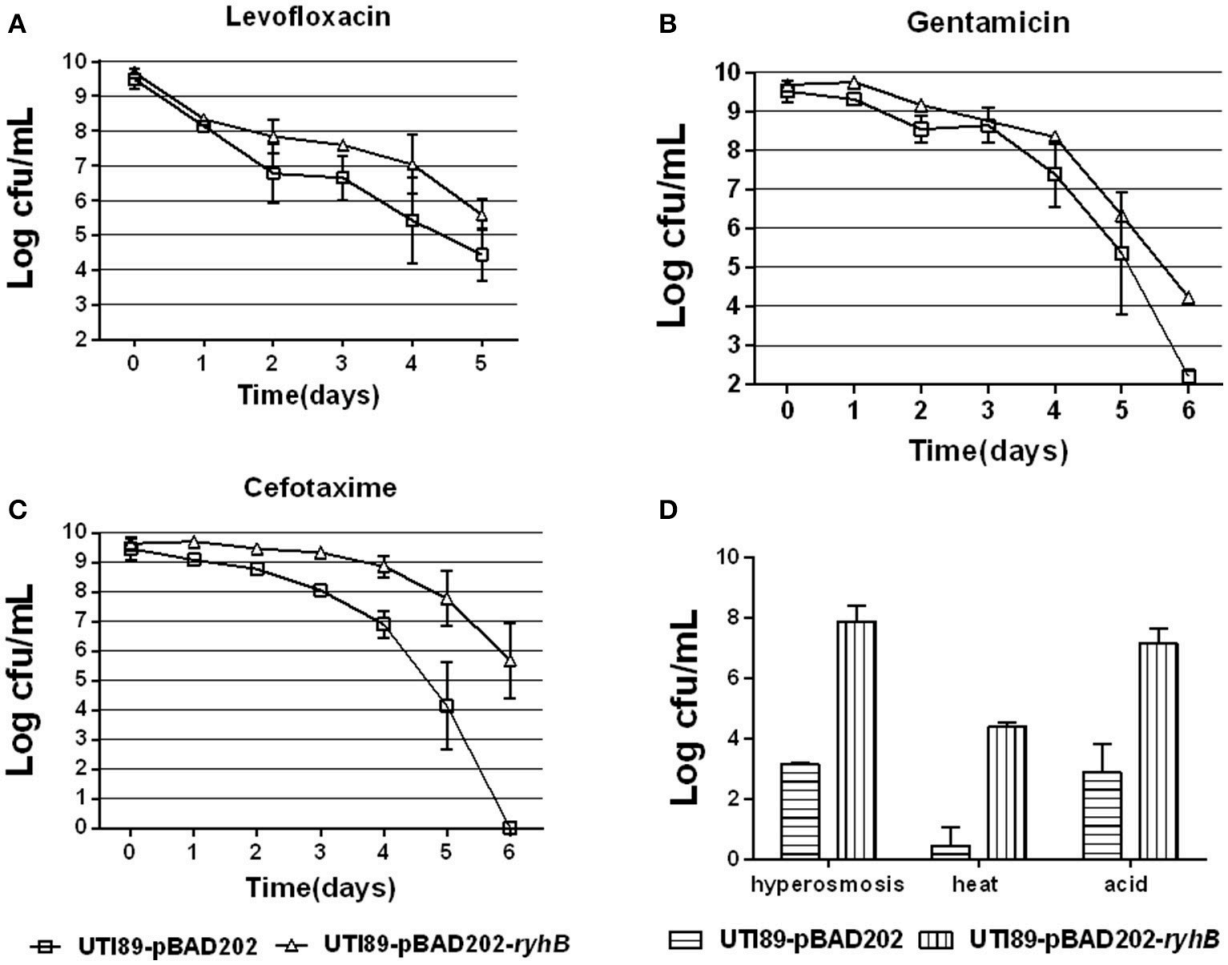
Overexpression of RyhB confers higher persistence levels. **(A–C)** Susceptibilities to antibiotics levofloxacin (5 μg/mL, **A**), gentamicin (30 μg/mL, **B**) or cefotaxime (128 μg/mL, **C**) at the time points indicated. **(D)** Susceptibilities to stresses hyperosmosis (NaCl, 4 M), heat (55°C) or acid for 3 d, 3 or 12 h, respectively. Bacteria were cultured to the stationary phase (~10^9^ CFU/mL) prior to the addition of antibiotics or stresses. Error bars show standard variations in CFU between at least three independent experiments.

To further determine if RyhB is involved in persister formation, we also overexpressed the RyhB in an inducible vector pBAD202-*ryhB* transformed into UTI89 and assessed their persister levels. We found a higher persister level in the RyhB overexpression strain than the parent strain carrying the empty vector pBAD202 at each antibiotic (levofloxacin, gentamicin, cefotaxime) and stress (hyperosmosis, heat, acid) we determined (Figure 3). As controls, we also determined the survival of all the strains in our experiments without any treatment, and found no difference at each timepoint (data not shown).

### RyhB confers persistence independent of Hfq

**Figure 4:**
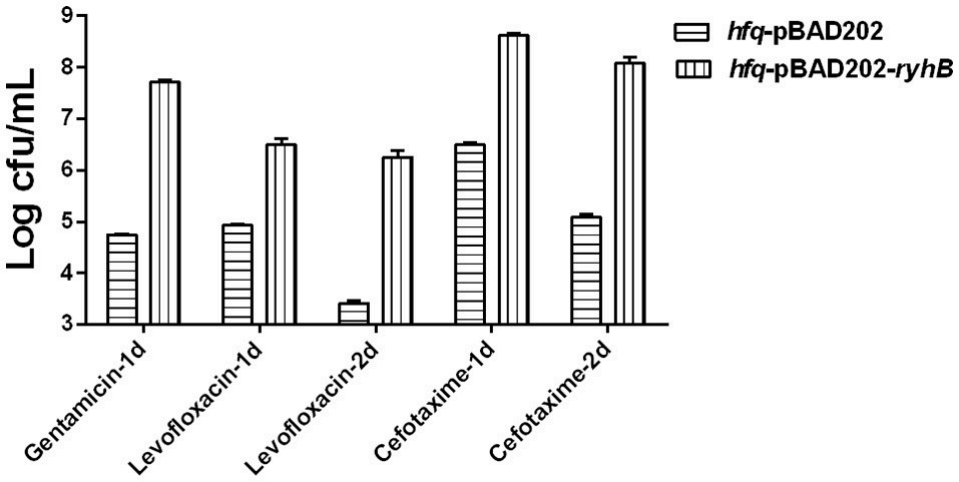
Effect of RyhB overexpression on persister formation in the *hfq*-knockout mutant. Antibiotics were added to stationary phase bacteria (~10^9^ CFU/mL) at the appropriate concentrations (gentamicin: 30 μg/mL; levofloxacin: 5 μg/mL; cefotaxime: 128 μg/mL). The number of persisters was counted at the time points indicated in the figure. Error bars show standard variations in CFU between at least three independent experiments. *hfq*-pBAD202, Hfq-knockout mutant carrying the empty vector pBAD202; *hfq*-pBAD202-*ryhB*, Hfq-knockout mutant carrying the *ryhB*-overexpression vector pBAD202-*ryhB*.

To determine whether Hfq was essential for the RyhB-specific persister phenotype, we transformed the RyhB-overexpression vector pBAD202-*ryhB* or the control empty vector, pBAD202 to an Hfq-knockout strain and subjected them to antibiotic exposure. The initial bacterial numbers were similar before antibiotics were added. The terminal numbers of the group without any treatment were similar too. Our results showed that the *ryhB*-overexpression strain was more tolerant to antibiotic challenge than the control strain in the absence of Hfq, with a ~10^3^-fold higher count of viable bacteria remaining following gentamicin (30 μg/mL), levofloxacin (5 μg/mL), or cefotaxime (128 μg/mL) exposure (Figure 4). These results suggest that RyhB-related bacterial persistence occurred independent of the Hfq protein. Additionally, we determined the susceptibilities of the Hfq-knockout strain to antibiotic challenge following exposure to gentamicin (30 μg/mL), levofloxacin (5 μg/mL), or cefotaxime (128 μg/mL). Compared with the parent strain, we observed defective persistence in the Hfq-knockout strain following exposure to each antibiotic (data not shown), which is consistent with a previous study (Kim and Wood, 2010).

### The effect of RyhB on persistence is independent of ppGpp

**Figure 5:**
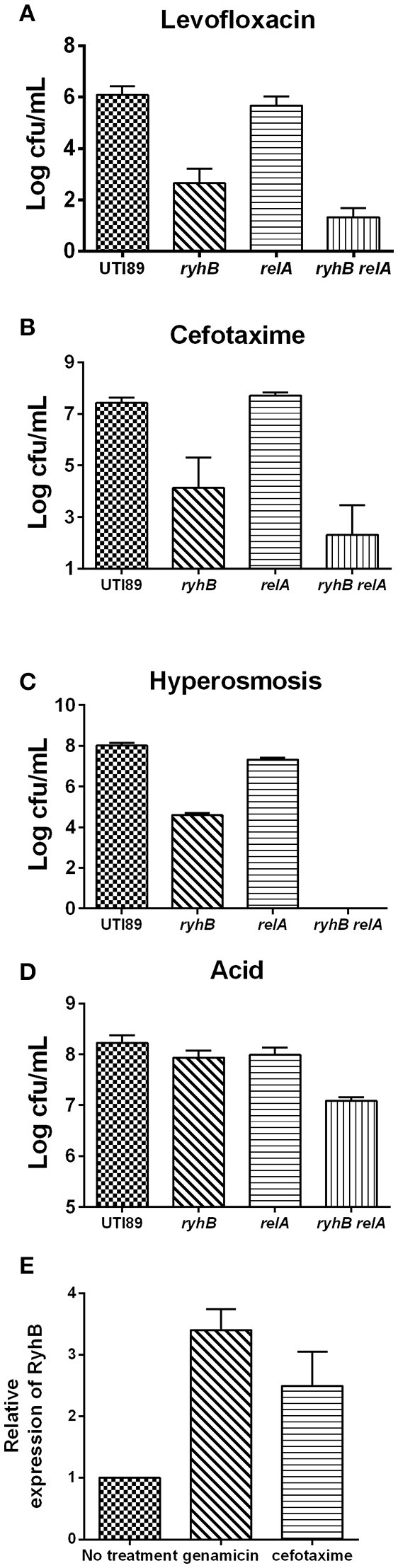
RyhB confers persistence in a ppGpp independent manner. ppGpp mutant strain Δ*relA* (*relA*), double-deletion mutant Δ*relA*Δ*ryhB* (*relA ryhB*), including the parent UTI89 and RyhB mutant strain Δ*ryhB* (*ryhB*), were grown to stationary phase (~10^9^ CFU/mL). Exposure pressures and incubation times are shown. **(A)** Susceptibilities to levofloxacin (5 μg/mL) at day 4. **(B)** Susceptibilities to cefotaxime (128 μg/mL) at day 4. **(C)** Susceptibilities to hyperosmosis (NaCl, 4 M) at day 1. **(D)** Susceptibilities to acid at pH 3.0 at 3 h. **(E)** Relative expression of RyhB in response to antibiotics. Stationary phase UTI89 bacteria were exposed to gentamicin (30 μg/mL) and cefotaxime (128 μg/mL) for 1 day, followed by extraction of total RNA for real-time PCR. Error bars show standard variations.

The first persistence gene, *hipA*, was shown to depend upon *relA*-dependent ppGpp synthesis to mediate persistence (Korch et al., 2003; Maisonneuve et al., 2013). To investigate whether RyhB-related persistence is also dependent upon ppGpp, we constructed a *relA*-deletion mutant, Δ*relA*, a RyhB mutant, Δ*ryhB*, and a double-deletion mutant, Δ*relA*Δ*ryhB*, and measured their persistence to different antibiotics and stresses. We observed that the number of surviving cells of the Δ*relA*Δ*ryhB* mutant was significantly lower than either of the two single mutants. The number of viable Δ*relA*Δ*ryhB* mutant cells decreased by ~10-fold following levofloxacin exposure (Figure 5A), ~10^2^-fold following cefotaxime exposure (Figure 5B), ~10^4^-fold during hyperosmotic stress (Figure 5C), and 5-fold upon exposure to acid pH (Figure 5D) as compared with either of the two single-mutant strains. These results indicate that the role of RyhB in persistence is independent of ppGpp, and that deletion of ppGpp further aggravated the persistence phenotype associated with Δ*ryhB* mutant. Interestingly, there was no apparent difference between the Δ*relA* mutant and the parent strain following antibiotic exposure, except that the cell number of the Δ*relA* mutant decreased 4-fold relative to that of the parent strain under hyperosmostic stress.

To investigate whether this phenomenon is unique to the uropathogenic *E. coli* UTI89 strain, we constructed a knockout strain, Δ*relA* in an *E. coli* K12 W3110 strain, and evaluated its effect on persistence. Our findings revealed that the W3110 Δ*relA* strain exhibited apparent defects in persistence as compared with the parent strain W3110 (data not shown). Therefore, these results indicated that the persistence-related role of *relA* in uropathogenic *E. coli* UTI89 differed from that in the *E. coli* K12 strain.

We then determined *ryhB*-expression level in the UTI89 strain upon challenging stationary phase bacteria with gentamicin (30 μg/mL) or cefotaxime (128 μg/mL) for 1 day (Figure 5E). Upon antibiotic exposure, we observed higher *ryhB*-expression levels than that in the untreated strain. The *ryhB*-expression levels were 3.4- and 2.4-fold relative to that of the parent strain upon exposure to gentamicin and cefotaxime, respectively. These results indicated that RyhB may be induced to protect cells in response to antibiotic stress.

### RyhB is synergistic with fur protein on persistence

**Figure 6:**
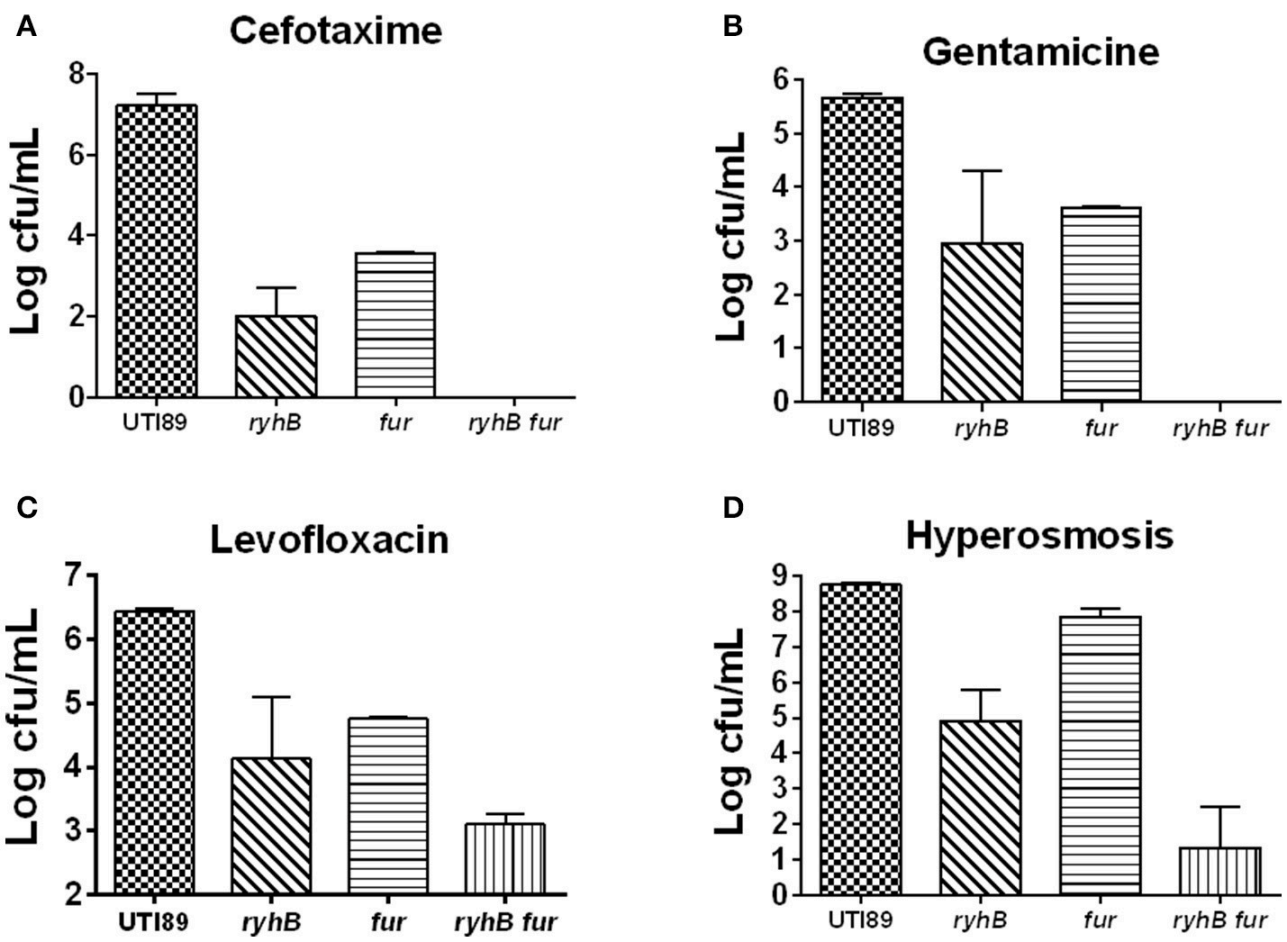
The relationship between RyhB and Fur. Fur-mutant strain Δ*fur (fur)*, double-deletion mutant Δ*ryhB*Δ*fur (ryhB fur)*, including the parent UTI89 and *ryhB*-mutant strain Δ*ryhB* (*ryhB*), were cultured for 10 h to the stationary phase prior to the addition of antibiotics or stresses. The types of antibiotics or stresses, as well as incubation times, are annotated. **(A)** Susceptibilities to cefotaxime (128 μg/mL) at day 5. **(B)** Susceptibilities to gentamicin (30 μg/mL) at day 4. **(C)** Susceptibilities to levofloxacin (5 μg/mL) at day 3. **(D)** Susceptibilities to hyperosmosis (NaCl, 4 M) at day 2. Error bars show standard variations in CFU between at least three independent experiments.

Since RyhB is regulated by the Fur repressor in the presence of iron, we wanted to address the relationship between Fur and RyhB on persistence. To test this, we constructed a single-mutant Δ*fur* strain and a double-mutant Δ*ryhB*Δ*fur* strain. We found that the number of surviving cells of the Δ*fur* mutant was more than 10^3^-fold lower than that in the parent strain upon exposure to cefotaxime (Figure 6A). Furthermore, we observed ~ 10^2^-fold lower viable cells following gentamicin (Figure 6B) or levofloxacin exposure (Figure 6C) and slightly lower levels (about 4-fold) following hyperosmosis (Figure 6D) in the Δ*fur* mutant than the parent strain. These results were contrary to our hypothesis, suggesting that Fur is unable to repress the RyhB-mediated persistence.

Additionally, we found that the Δ*ryhB*Δ*fur* mutant had >10^2^ times lower CFU than either that of the single-mutants Δ*ryhB* or Δ*fur* following exposure to cefotaxime or gentamicin, as well as hyperosmosis (Figures 6A,B,D). Furthermore, we observed ~10-fold lower cell survival following exposure to levofloxacin (Figure 6C) in the double mutant than that of either of the single mutants. These results indicated that the persister phenotype associated with the Δ*ryhB*Δ*fur* double mutant was more pronounced than that of either of the single Δ*ryhB* or Δ*fur* mutant, suggesting that RyhB exhibits a synergistic effect with Fur in persister formation.

### RNA-seq analysis sheds new light on the mechanisms of RyhB-mediated persistence

Because our data indicated RyhB involvement in persistence, we wanted to investigate how RyhB is involved in persister formation. Therefore, we analyzed genes regulated by RyhB by comparing the Δ*ryhB* mutant and the parent strain UTI89 through RNA-Seq analysis. We found that ~200 genes were upregulated by at least ≥2-fold in the Δ*ryhB* mutant strain, whereas 130 genes were downregulated compared to the parent UTI89 strain. The upregulated genes were mainly comprised of genes belonging to cell motility, transport, toxin-antitoxin modules, DNA repair, transcription, and metabolism (Table 1).

**Table 1:** Genes upregulated by ≥2-fold in the *ryhB*-knockout mutant relative to the parent strain UTI89 according to RNA-Seq analysis.

**Description**	**Gene**
Cell motility	*flgC, -D*
	*malE*
Transporter systems	*fepC*
	*ycbO*
	*proW*
	*malK, -M*
DNA repair	*dinI*
	*umuC, -D*
	*yebG*
	*recN*
Two-component systems	*iroN*
TA modules	*relE*
	*yoeB*
Transcriptional regulators	*traL*
	*ygaA, -V*
	*yqjI*
	*dsdC*
	*tqsA*
	*lamB*
	*soxS*
	*cpxP*
	*yhhY*
Metabolic enzymes	*sdhA, -B, -C, -D*
	*carB*
	*yeiA*
	*ubiF*
	*prpB, -C, -D*
	*yeiT*
	*trpD*
	*cpsG*
	*rhaB*
	*ycjM*

### RyhB deletion results in a metabolically hyperactive state

**Figure 7:**
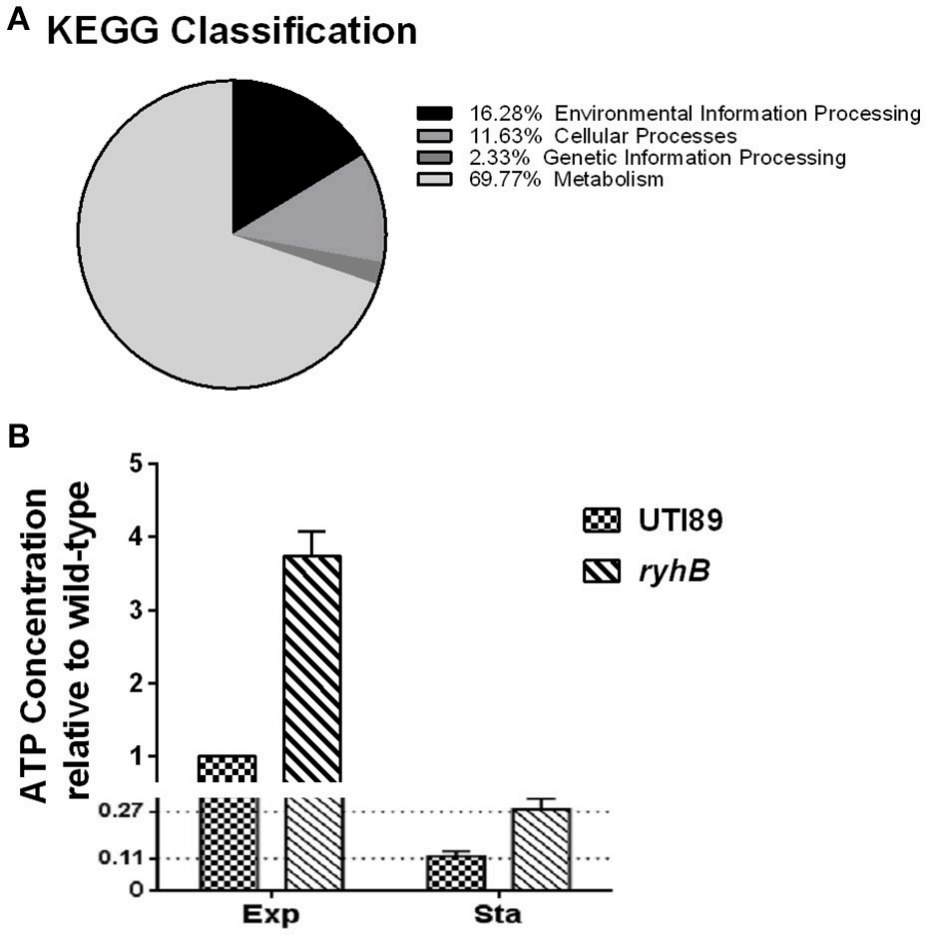
The *ryhB*-knockout mutant causes bacterial transition to a hyperactive metabolic state. **(A)** KEGG classification of upregulated genes in the *ryhB*-knockout mutant. Stationary phase bacteria (cultured for 10 h, ~10^9^ CFU/mL) were used for RNA-Seq analysis. **(B)** Relative ATP concentration in the parent strain UTI89 and the *ryhB*-knockout mutant at the exponential (Exp, cultured for 3 h, ~10^8^ CFU/mL) and stationary phases (Sta, cultured for 10 h, ~10^9^ CFU/mL). ATP concentration of the parent strain during the exponential phase was considered as 1 unit. Error bars show standard variations (*n* = 3).

Among elevated genes in the Δ*ryhB* mutant strain, metabolic genes accounted for ~70% of the total genes according to Kyoto Encyclopedia of Genes and Genomes (KEGG) classification (Figure 7A). For this reason, we hypothesized that the Δ*ryhB* mutant may lead to a metabolically hyperactive state. To test this hypothesis, we determined cellular ATP levels which represent the major energy currency of cells. If the strain is in a metabolically hyperactive state, the ATP level should be high. As expected, the ATP level of the *ryhB*-knockout strain was over 2-fold higher than that of the parent strain in both exponential and stationary phase cultures (Figure 7B).

To further confirm that RyhB deletion resulted in a metabolically hyperactive state, intracellular NAD^+^/NADH ratios were measured (Maeda et al., 2017). We found Δ*ryhB* mutant showed reduced NAD^+^/NADH ratios in both exponential (2.76-fold decrease) and stationary phase (3.66-fold decrease) cultures (Table 2) when compared with the parent strain. These results indicate that the intracellular environment of the Δ*ryhB* mutant was more reductive compared with the parent strain, suggesting the Δ*ryhB* mutant caused a metabolically hyperactive state.

**Table 2:** Intracellular NAD^+^/NADH ratios of the parent strain UTI89 and the Δ*ryhB* mutant.

**Culture phase**	**Ratio (NAD^+^/NADH)^a^**		**Ratio (UTI89/Δ*ryhB*)**
	**UTI89**	**Δ*ryhB***	
Exp	24.43 ± 1.24	8.84 ± 0.46	2.76
Sta	17.67 ± 3.61	4.83 ± 0.15	3.66

*NAD^+^/NADH ratios in the parent strain UTI89 and the ΔryhB mutant at the exponential (Exp, cultured for 3 h, ~10^8^ CFU/mL) and stationary phases (Sta, cultured for 10 h, ~10^9^ CFU/mL)*.

a *Values are means ± standard deviations (n = 3)*.

## Discussion

Despite numerous studies investigating the persister phenomenon, the mechanisms of persister formation remain poorly understood. The versatile and complex nature of persister formation prompted us to examine the possible roles of sRNAs in persister formation, given their roles in persistence remain unexplored. In this study, we identified RyhB as a critical sRNA being involved in persister formation for the first time, as mutation in RyhB led to decreased persister numbers to different classes of antibiotics and stresses (Figures 1–3). RNA-Seq analysis indicated that RyhB knockout caused a hyperactive metabolic state, which is confirmed by elevated ATP levels and altered NAD^+^/NADH ratios in the *ryhB*-knockout mutant.

RyhB is an important sRNA to regulate iron consumption and storage under Fe^2+^ depletion conditions through base-pairing mechanism (Masse and Gottesman, 2002). During iron limitation, Fur dissociates from the *ryhB* operon, leading to the expression of RyhB. RyhB can repress iron-utilizing non-essential mRNA transcription which makes iron available for essential proteins. RyhB can also regulate siderophore production and virulence (Porcheron et al., 2014) and control the redox state of anaerobic metabolism in *Enterobacter aerogenes* (Wu et al., 2017). In this study, we found that the RyhB-deletion mutant showed defects in persister-formation capacity against antibiotics and stresses (Figures 1–3) and identified a new role for *ryhB* as a new persister gene. *ryhB* deletion resulted in upregulation of multiple genes involved in persister-related pathways (Table 1), causing the bacteria to transition to a hyperactive metabolic state. Because cells are more susceptible to antibiotics and stresses in an active metabolic state, RyhB deletion mutant showed defects in persister formation. This finding is reminiscent of our previous observation of the *phoU* mutant which has a profound defect in persistence and has a hyperactive metabolic state (Li and Zhang, 2007). A previous report showed that metabolic stimulation can be used to eradicate persisters (Allison et al., 2011b), indicating that inhibition of sRNAs (RyhB) might be another alternative method for eliminating persisters. In addition, previous studies showed ATP depletion can suppress the activities of antibiotic targets facilitating persister formation (Conlon et al., 2016; Shan et al., 2017). Our finding of RyhB mutation causing defect in persisters with elevated ATP production is consistent with this observation in *E. coli*.

Hfq protein is known to regulate sRNAs in many bacteria. This protein can protect sRNAs from degradation and prompt sRNA to match with targets (Updegrove et al., 2016). However, in our study, we found the persister role of RyhB was independent of Hfq. The strain harboring Hfq protein did have a higher persister level than the strain without Hfq protein when RyhB was overexpressed (Figures 3, 4). In the absence of Hfq, the overexpression of RyhB could also cause increased persister level (Figure 4). This indicates that RyhB involvement in persistence is not dependent on Hfq. This is consistent with the previous study that showed RyhB could also regulate target SodB in Hfq deletion background (Geissmann and Touati, 2004).

As a major stringent-response regulator of *E. coli* K12, RelA represents the primary ppGpp synthetase, whose deficiency generates fewer persister cells (Korch et al., 2003). In our study, we did not observe a significant difference between the Δ*relA* mutant and the parent strain UTI89 (Figure 5). This might have been due to the following possibilities: (1) the function of *relA* in the UTI89 strain might not be similar with that of *E. coli* K12 strains. Because we found that there are 49 nucleotide differences in the *relA* sequences between the clinical UTI89 strain and the laboratory *E. coli* K12 stains. (2) As a clinical UTI strain, UTI89 is more tolerant to external adverse environments than laboratory *E. coli* K12 strains. For example, deletion of *rpoE* in the UTI89 strain was not as lethal as in laboratory *E. coli* K12 strains (Button et al., 2007). Additionally, the UTI89 strain showed greater tolerance to reactive oxygen species and reactive nitrogen species than *E. coli* K12 strains (Rama et al., 2005; Kulesus et al., 2008). In this study, the Δ*relA*Δ*ryhB* double mutant showed more obvious persister defect phenotype than either of the single mutant. This observation indicates that RelA exhibited a synergic effect with RyhB in persister formation, which is consistent with the previous observation that RelA protein stimulated the RyhB activity (Argaman et al., 2012).

In summary, this study represents the first report demonstrating that sRNA plays important roles in persister formation using a clinically relevant uropathogenic strain of *E. coli* UTI89. The sRNA RyhB plays a major role in persistence in a manner that is independent of the sRNA-binding protein Hfq, but synergistic with the ppGpp and Fur proteins. Further studies are needed to determine whether sRNA RyhB is involved in *in vivo* persistence in animal models. Because sRNAs represent a quick and efficient way to regulate gene expression without protein synthesis, they could play a versatile role in rapid persister formation in response to environmental stresses or antibiotic exposure. Our findings expand the current understanding of mechanisms of persistence to the sRNA level and may have implications for developing improved treatment of persistent bacterial infections.

## Author contributions

YZ and WZ designed the experiments; SZ and SL completed all the experiments, SZ, NW and YY performed the data analysis; SZ and YZ wrote the manuscript.

### Conflict of interest statement

The authors declare that the research was conducted in the absence of any commercial or financial relationships that could be construed as a potential conflict of interest.
