# Supplementary material for: Small Non-coding RNA RyhB Mediates Persistence to Multiple Antibiotics and Stresses in Uropathogenic Escherichia coli by Reducing Cellular Metabolism
Source: Front Microbiol. 2018 Feb 6;9:136. doi: 10.3389/fmicb.2018.00136 (PMC5808207; doi:10.3389/fmicb.2018.00136)
Supplement: Supplementary file 3 [file Table3.DOCX]

Supplementary Material

**Small non-coding RNA RyhB mediates persistence to multiple antibiotics and stresses in** **uropathogenic *Escherichia coli* by reducing cellular metabolism**

**Shanshan Zhang^1^, Shuang Liu^1^, Nan Wu^1^, Youhua Yuan^1^, Wenhong Zhang^1^* and Ying Zhang^1, 2^***

^1^ Key Lab of Molecular Virology, Department of Infectious Diseases, Huashan Hospital, Fudan University, Shanghai, China,

^2^ Department of Molecular Microbiology and Immunology, Bloomberg School of Public Health, Johns Hopkins University, Baltimore, MD, USA

*** Correspondence:**Ying Zhang
yzhang@jhsph.edu

Wenhong Zhang
zhangwenhong@fudan.edu.cn

# Supplementary Table 3. Primers used for real-time PCR.

| **Gene** | **Primer (5′–3′)** |
| --- | --- |
| *ryhB* | F: AGAACCTGAAAGCACGACATTG  R: GGCTGGCTAAGTAATACTGGAAG |
| *rrsB* | F: ATGGCTCAGATTGAACGC  R: GGCAGTTTCCCAGACATTAC |
